# Supplementary material for: Comparison of the Effect of Phospholipid Extracts from Salmon and Silver Carp Heads on High-Fat-Diet-Induced Metabolic Syndrome in C57BL/6J Mice
Source: Mar Drugs. 2023 Jul 19;21(7):409. doi: 10.3390/md21070409 (PMC10381321; doi:10.3390/md21070409)
Supplement: Supplementary file 1 [file marinedrugs-21-00409-s001.zip › marinedrugs-2487390-supplementary.pdf]

Table S1 Primer sequences for RT-qPCR amplification

Gene	Forward primer	Reverse primer
FAS	TTGATGATTCAGGGAGTGGA	AGCAGATGAGTTGTTCTTGGAC
ME	TCACCTGCCCTAATGTCCCT	CATGCCGTTATCAACTTGTCC
G6PDH	GTTTGGCAGCGGCAACTAA	GGCATCACCCTGGTACAACTC
ACC	GAGTGACTGCCGAAACATCTCTG	GCAAGGAGGACAGAGTTTATCGTG
PPARα	GTACGGCAATGGCTTTATCA	CAATCCCCTCCTGCAACTT
CPT-1a	CTCAGTGGGAGCGACTCTTCA	GGCCTCTGTGGTACACGACAA
CPT-2	GCTCCGAGGCATTTGTCA	CCCATCGCTGCTTCTTTG
PPARγ	GTGATGGAAGACCACTCGC	CCCACAGACTCGGCACTC
IRS1	TTGCTTGGCACAATGTAGAA	GAGGATCGTCAATAGCGTAAC
PI3Kr1	CCTCTCCTTATAAAGCTCCTGGAA	GATCACAATCAAGAAGCTGTCGTAA
Akt1	ACACGATGTTGGCAAAGAA	GTGCTGGAGGACAACGACT
Akt2	TTCTACAACCAGGACCACGAGC	TGATGCTGAGGAAGAACCGATG
GSK-3β	AACACCAACAAGGGAGCA	GAGCGTGAGGAGGGATAA
GYS2	ATCAGGCTTCCTCTTCAGCA	CCAGCTTGATAAGTTCAACA
GLUT2	CTGTCTGTGTCCAGCTTTGCA	CAAGCCACCCACCAAAGAAC
GAPDH	CATCACTGCCACCCAGAAGACTG	ATGCCAGTGAGCTTCCCGTTCAG

Table S2. PL Molecular species of SC-PLE determined by (ultrahigh-performance liquid chromatography-quadrupole-time-off light mass spectrometry) UHPLC-Q-TOF-MS.

Molecular Species	RT (min)	m/z	Precursors	Contents/(nmol/g)
LPA 18:0	2.21	437.2645	[M-H]-	3308.26
LPA 18:1	1.66	435.2466	[M-H]-	1493.15
LPA 20:3	1.45	459.2459	[M-H]-	96.80
LPA 20:5	1.12	455.2160	[M-H]-	399.99
LPA 22:6	1.18	481.2368	[M-H]-	2453.60
LPC 14:0	1.76	526.3146	[M+CH3COO]-	4520.84
LPC 15:0	2.13	540.3259	[M+CH3COO]-	2772.01
LPC 16:0	2.46	554.3438	[M+CH3COO]-	66594.72
LPC 17:0	2.74	568.3516	[M+CH3COO]-	3241.90
LPC 17:1	2.31	566.3446	[M+CH3COO]-	2038.69
LPC 18:0	3.09	582.3766	[M+CH3COO]-	15556.50
LPC 18:1	2.56	580.3594	[M+CH3COO]-	12320.18
LPC 18:2	2.18	578.3453	[M+CH3COO]-	3215.19
LPC 18:3	1.73	576.3293	[M+CH3COO]-	2277.22
LPC 18:4	1.42	574.3149	[M+CH3COO]-	320.19
LPC 20:0	3.72	610.3990	[M+CH3COO]-	239.65
LPC 20:1	3.15	608.3921	[M+CH3COO]-	1117.29
LPC 20:2	2.75	606.3660	[M+CH3COO]-	537.73
LPC 20:3	2.38	604.3613	[M+CH3COO]-	1099.25
LPC 20:4	2.08	602.3429	[M+CH3COO]-	4078.62
LPC 20:5	1.68	600.3339	[M+CH3COO]-	6579.32
LPC 22:0	4.34	638.4308	[M+CH3COO]-	72.56
LPC 22:1	3.77	636.4227	[M+CH3COO]-	231.49
LPC 22:3	2.96	632.3856	[M+CH3COO]-	103.72
LPC 22:5	2.38	628.3553	[M+CH3COO]-	3121.30
LPC 22:6	2.03	626.3466	[M+CH3COO]-	12626.31
LPC 24:0	5.03	666.4609	[M+CH3COO]-	129.54
LPC 24:1	4.37	664.4493	[M+CH3COO]-	469.19
LPE 14:0	1.63	424.2442	[M-H]-	17.00
LPE 16:0	2.32	452.2765	[M-H]-	1012.65
LPE 16:1	1.81	450.2569	[M-H]-	126.94
LPE 17:0	2.60	466.2923	[M-H]-	196.15
LPE 18:0	2.89	480.3051	[M-H]-	1893.34
LPE 18:1	2.46	478.2923	[M-H]-	1261.22
LPE 18:2	2.07	476.2751	[M-H]-	137.59
LPE 18:3	1.62	474.2632	[M-H]-	38.54
LPE 19:0	3.15	494.3205	[M-H]-	92.98
LPE 20:1	2.98	506.3191	[M-H]-	142.45
LPE 20:2	2.62	504.3075	[M-H]-	56.33
LPE 20:3	2.27	502.2936	[M-H]-	105.25
LPE 20:4	2.00	500.2755	[M-H]-	566.01

LPE 20:5	1.57	498.2635	[M-H]-	341.78
LPE 22:5	2.17	526.2913	[M-H]-	138.22
LPE 22:5	2.29	526.2922	[M-H]-	573.52
LPE 22:6	1.95	524.2863	[M-H]-	2469.72
LPE 24:1	3.77	562.3878	[M-H]-	10.31
LPE 26:1	4.37	590.4098	[M-H]-	19.55
LPG 16:0	1.35	483.2687	[M-H]-	423.37
LPG 18:0	2.04	511.3008	[M-H]-	132.67
LPG 18:1	1.49	509.2861	[M-H]-	256.62
LPG 18:2	1.13	507.2667	[M-H]-	23.01
LPG 20:1	2.14	537.3194	[M-H]-	59.03
LPG 22:5	1.34	557.2867	[M-H]-	65.01
LPG 22:6	1.08	555.2730	[M-H]-	115.12
LPI 18:2	1.12	595.2835	[M-H]-	38.98
LPI 20:5	1.04	617.2740	[M-H]-	64.19
LPS 16:0	1.27	496.2646	[M-H]-	1227.70
LPS 17:0	1.57	510.2822	[M-H]-	859.63
LPS 18:1	1.42	522.2833	[M-H]-	1561.55
LPS 18:2	1.12	520.2672	[M-H]-	156.44
LPS 19:0	2.25	538.3049	[M-H]-	392.40
LPS 20:1	2.08	550.3082	[M-H]-	245.35
LPS 20:3	1.24	546.2790	[M-H]-	198.85
LPS 20:4	1.12	544.2689	[M-H]-	340.34
LPS 22:4	1.43	572.2968	[M-H]-	410.81
LPS 22:5	1.26	570.2800	[M-H]-	1918.05
LPS 22:6	1.08	568.2690	[M-H]-	2019.80
PA 16:0_18:1	4.34	673.4786	[M-H]-	546.23
PA 16:1_18:1	4.10	671.4606	[M-H]-	224.92
PA 18:1_18:1	4.40	699.4922	[M-H]-	415.24
PA 18:1_18:2	4.17	697.4762	[M-H]-	210.02
PA 16:0_20:4	4.04	695.4604	[M-H]-	553.58
PA 16:0_20:5	3.85	693.4426	[M-H]-	463.52
PA 17:0_20:4	4.20	709.4709	[M-H]-	175.66
PA 17:0_20:5	4.00	707.4637	[M-H]-	128.23
PA 18:0_20:3	4.53	725.5118	[M-H]-	203.95
PA 18:0_20:4	4.37	723.4962	[M-H]-	1761.04
PA 18:0_20:5	4.17	721.4770	[M-H]-	2109.89
PA 16:0_22:6	3.98	719.4623	[M-H]-	1435.12
PA 16:1_22:6	3.71	717.4453	[M-H]-	164.92
PA 17:0_22:6	4.13	733.4760	[M-H]-	257.99
PA 18:0_22:5	4.40	749.5072	[M-H]-	981.98
PA 18:0_22:6	4.31	747.4948	[M-H]-	3272.34
PA 18:1_22:6	4.01	745.4832	[M-H]-	455.40
PA 20:4_20:4	3.79	743.4589	[M-H]-	199.00

PA 20:4_20:5	3.61	741.4498	[M-H]-	170.72
PA 20:5_22:6	3.57	765.4448	[M-H]-	129.82
PA 20:4_22:5	3.87	769.4896	[M-H]-	204.22
PA 22:5_22:5	3.87	795.4995	[M-H]-	476.09
PA 22:6_22:6	3.71	791.4654	[M-H]-	882.17
PC 16:0_16:0	5.95	792.5757	[M+CH3COO]-	15261.96
PC 16:0_17:0	6.33	806.5827	[M+CH3COO]-	3649.84
PC 16:0_17:1	5.69	804.5696	[M+CH3COO]-	6937.07
PC 16:1_17:1	5.17	802.5498	[M+CH3COO]-	1005.95
PC 16:0_18:0	6.82	820.6068	[M+CH3COO]-	4010.28
PC 16:0_18:1	6.01	818.5927	[M+CH3COO]-	71264.94
PC 16:1_18:1	5.55	816.5749	[M+CH3COO]-	9079.34
PC 16:0_18:3	5.16	814.5580	[M+CH3COO]-	2861.06
PC 16:0_18:4	4.88	812.5461	[M+CH3COO]-	1258.09
PC 16:0_19:0	7.30	834.6366	[M+CH3COO]-	941.10
PC 17:0_18:1	6.46	832.6054	[M+CH3COO]-	8145.92
PC 17:1_18:1	5.82	830.5847	[M+CH3COO]-	1529.74
PC 15:0_20:5	4.78	824.5410	[M+CH3COO]-	682.71
PC 18:0_18:1	6.87	846.6299	[M+CH3COO]-	26337.92
PC 18:1_18:1	6.10	844.6022	[M+CH3COO]-	3489.85
PC 16:0_20:4	5.36	840.5759	[M+CH3COO]-	12158.01
PC 16:0_20:5	5.02	838.5565	[M+CH3COO]-	27261.98
PC 19:0_18:1	7.37	860.6374	[M+CH3COO]-	1577.36
PC 18:1_19:1	6.55	858.6235	[M+CH3COO]-	837.18
PC 15:0_22:6	4.96	850.5557	[M+CH3COO]-	1453.87
PC 18:0_20:3	6.45	870.6155	[M+CH3COO]-	935.85
PC 16:0_22:4	5.86	868.6097	[M+CH3COO]-	1706.77
PC 18:0_20:4	6.01	868.5986	[M+CH3COO]-	2069.77
PC 16:0_22:5	5.44	866.5910	[M+CH3COO]-	5068.80
PC 18:0_20:5	5.59	866.5908	[M+CH3COO]-	12633.05
PC 16:0_22:6	5.22	864.5745	[M+CH3COO]-	24354.76
PC 18:3_20:5	4.42	860.5432	[M+CH3COO]-	721.84
PC 17:0_22:5	5.83	880.6003	[M+CH3COO]-	1033.74
PC 17:0_22:6	5.47	878.5873	[M+CH3COO]-	2323.87
PC 24:0_16:1	8.85	902.6836	[M+CH3COO]-	2949.97
PC 16:1_24:1	7.86	900.6649	[M+CH3COO]-	2365.49
PC 18:0_22:5	6.34	894.6130	[M+CH3COO]-	1339.37
PC 18:0_22:6	5.83	892.6053	[M+CH3COO]-	5373.96
PC 24:0_18:1	9.92	930.7189	[M+CH3COO]-	4193.31
PC 20:5_22:6	4.49	910.5566	[M+CH3COO]-	708.11
PC 18:1_24:1	8.80	928.6990	[M+CH3COO]-	9677.02
PC 24:1_18:2	8.06	926.6840	[M+CH3COO]-	703.26
PC 20:1_22:6	5.85	918.6251	[M+CH3COO]-	465.96
PC 20:3_22:6	5.06	914.5856	[M+CH3COO]-	784.83

PC 26:0_18:1	11.19	958.7471	[M+CH3COO]-	829.66
PC 18:1_26:1	9.89	956.7286	[M+CH3COO]-	3740.28
PC 26:1_18:2	9.06	954.7031	[M+CH3COO]-	524.92
PC 24:1_20:3	8.33	952.6973	[M+CH3COO]-	287.82
PC 24:0_20:5	8.05	950.6872	[M+CH3COO]-	142.19
PC 18:1_28:1	11.09	984.7576	[M+CH3COO]-	780.14
PC O-16:0_16:0;3O	5.85	826.5936	[M+CH3COO]-	5781.42
PC O-16:0_16:1;3O	5.44	824.5795	[M+CH3COO]-	10671.70
PC O-17:0_16:1;3O	5.66	838.5854	[M+CH3COO]-	1154.25
PC O-16:0_18:0;3O	6.49	854.6267	[M+CH3COO]-	940.54
PC O-16:0_18:1;3O	6.15	852.6082	[M+CH3COO]-	6675.05
PC O-16:0_18:2;3O	5.68	850.5930	[M+CH3COO]-	27017.35
PC O-17:0_18:2;3O	5.89	864.6104	[M+CH3COO]-	3331.43
PC O-18:0_18:2;3O	6.45	878.6247	[M+CH3COO]-	3525.32
PC O-18:1_18:2;3O	5.73	876.6036	[M+CH3COO]-	3443.01
PC O-18:3_18:2;3O	4.93	872.5781	[M+CH3COO]-	1437.01
PE 16:0_16:1	4.93	688.4870	[M-H]-	150.76
PE 16:0_17:0	5.63	704.5206	[M-H]-	157.44
PE 15:0_18:1	5.17	702.5054	[M-H]-	131.28
PE 16:0_18:1	5.42	716.5223	[M-H]-	1501.02
PE 16:1_18:1	5.00	714.5049	[M-H]-	310.00
PE 16:1_18:2	4.72	712.4893	[M-H]-	42.06
PE 17:0_18:0	6.36	732.5558	[M-H]-	174.16
PE 17:0_18:1	5.68	730.5352	[M-H]-	454.72
PE 17:1_18:1	5.24	728.5150	[M-H]-	107.03
PE 18:0_18:0	6.82	746.5640	[M-H]-	148.99
PE 18:0_18:1	5.98	744.5537	[M-H]-	4973.27
PE 18:1_18:1	5.52	742.5387	[M-H]-	1282.27
PE 18:1_18:2	5.17	740.5193	[M-H]-	317.67
PE 16:0_20:4	4.96	738.5035	[M-H]-	826.65
PE 16:0_20:5	4.68	736.4954	[M-H]-	482.00
PE 17:0_20:1	6.48	758.5687	[M-H]-	393.40
PE 18:1_19:1	5.81	756.5423	[M-H]-	115.82
PE 17:0_20:4	5.20	752.5212	[M-H]-	293.60
PE 18:0_20:1	6.88	772.5836	[M-H]-	846.38
PE 18:1_20:1	6.05	770.5707	[M-H]-	603.87
PE 18:0_20:4	5.46	766.5369	[M-H]-	4611.17
PE 18:1_20:4	5.02	764.5202	[M-H]-	1819.41
PE 16:0_22:6	4.86	762.5057	[M-H]-	1277.18
PE 18:1_20:5	4.74	762.5126	[M-H]-	386.47
PE 19:0_20:5	5.39	778.5287	[M-H]-	444.17
PE 17:0_22:6	5.06	776.5226	[M-H]-	415.32
PE 22:0_18:1	7.19	800.6088	[M-H]-	48.49
PE 18:1_22:1	6.60	798.5944	[M-H]-	73.03

PE 18:0_22:4	5.86	794.5664	[M-H]-	564.92
PE 18:0_22:5	5.68	792.5501	[M-H]-	2355.14
PE 18:0_22:6	5.35	790.5381	[M-H]-	5880.99
PE 18:1_22:5	5.22	790.5488	[M-H]-	877.92
PE 18:1_22:6	4.91	788.5240	[M-H]-	913.92
PE 18:2_22:6	4.59	786.5014	[M-H]-	142.07
PE 20:4_20:5	4.33	784.4897	[M-H]-	66.04
PE 19:1_22:6	5.17	802.5413	[M-H]-	167.66
PE 20:5_22:6	3.23	808.4915	[M-H]-	35.73
PE 18:1_24:1	7.21	826.6301	[M-H]-	87.69
PE 20:3_22:5	5.00	814.5382	[M-H]-	201.39
PE 20:4_22:5	4.71	812.5217	[M-H]-	180.01
PE 22:6_22:6	4.42	834.5081	[M-H]-	307.69
PE O-16:1_14:0	5.13	646.4767	[M-H]-	49.78
PE O-16:1_15:0	5.37	660.4943	[M-H]-	64.82
PE O-16:0_16:0	5.86	676.5255	[M-H]-	51.42
PE O-16:1_16:0	5.66	674.5118	[M-H]-	386.21
PE O-16:1_16:1	5.20	672.4974	[M-H]-	843.01
PE O-18:2_14:1	4.80	670.4715	[M-H]-	23.99
PE O-16:1_17:0	5.86	688.5226	[M-H]-	271.97
PE O-16:1_17:1	5.46	686.5120	[M-H]-	771.23
PE O-18:2_15:1	5.04	684.4962	[M-H]-	37.84
PE O-18:0_16:0	6.70	704.5617	[M-H]-	144.41
PE O-18:1_16:0	6.24	702.5430	[M-H]-	606.77
PE O-16:1_18:1	5.72	700.5262	[M-H]-	6307.57
PE O-18:2_16:1	5.27	698.5123	[M-H]-	1037.70
PE O-16:1_18:3	4.99	696.4864	[M-H]-	82.57
PE O-17:1_18:1	6.01	714.5424	[M-H]-	1175.70
PE O-18:2_17:1	5.53	712.5267	[M-H]-	495.29
PE O-15:1_20:4	4.97	708.4913	[M-H]-	95.46
PE O-18:0_18:1	6.72	730.5718	[M-H]-	627.90
PE O-18:1_18:1	6.30	728.5576	[M-H]-	4805.83
PE O-18:2_18:1	5.79	726.5406	[M-H]-	4350.49
PE O-18:3_18:1	5.40	724.5175	[M-H]-	408.93
PE O-16:1_20:4	5.22	722.5140	[M-H]-	2714.78
PE O-16:1_20:5	4.91	720.4954	[M-H]-	778.70
PE O-14:1_22:6	4.63	718.4792	[M-H]-	50.40
PE O-19:1_18:1	6.54	742.5734	[M-H]-	187.94
PE O-19:2_18:1	6.05	740.5569	[M-H]-	158.50
PE O-17:1_20:4	5.42	736.5261	[M-H]-	817.81
PE O-17:1_20:5	5.06	734.5074	[M-H]-	385.38
PE O-15:1_22:6	4.86	732.4874	[M-H]-	126.37
PE O-18:1_20:1	6.91	756.5873	[M-H]-	266.37
PE O-18:2_20:1	6.36	754.5696	[M-H]-	245.40

PE O-18:0_20:4	5.86	752.5558	[M-H]-	528.35
PE O-18:1_20:4	5.75	750.5422	[M-H]-	2086.41
PE O-16:1_22:5	5.26	748.5280	[M-H]-	3108.53
PE O-16:1_22:6	5.09	746.5143	[M-H]-	5111.33
PE O-16:2_22:6	4.71	744.4929	[M-H]-	120.34
PE O-19:1_20:4	5.91	764.5544	[M-H]-	282.72
PE O-17:1_22:5	5.44	762.5435	[M-H]-	357.74
PE O-17:2_22:6	4.93	758.5032	[M-H]-	59.18
PE O-18:4_22:6	4.52	768.5017	[M-H]-	55.50
PE O-18:1_22:5	5.97	776.5647	[M-H]-	2266.92
PE O-18:1_22:6	5.60	774.5460	[M-H]-	4399.33
PE O-18:2_22:6	5.16	772.5307	[M-H]-	1598.65
PE O-18:3_22:6	4.80	770.5105	[M-H]-	152.68
PE O-19:1_22:6	5.85	788.5548	[M-H]-	346.68
PE O-19:2_22:6	5.39	786.5376	[M-H]-	90.18
PE O-20:4_22:6	4.91	796.5444	[M-H]-	120.62
PE O-20:1_22:6	5.76	802.5699	[M-H]-	175.11
PG 16:0_18:1	4.09	747.5131	[M-H]-	228.47
PG 16:1_18:1	3.88	745.4954	[M-H]-	30.69
PG 18:0_18:1	4.39	775.5450	[M-H]-	101.58
PG 18:1_18:1	4.15	773.5308	[M-H]-	80.50
PG 16:0_20:3	3.97	771.5155	[M-H]-	82.41
PG 18:1_18:3	3.75	769.4960	[M-H]-	36.72
PG 18:0_20:2	4.43	801.5626	[M-H]-	14.61
PG 18:1_20:2	4.24	799.5449	[M-H]-	28.89
PG 16:0_22:6	3.81	793.5018	[M-H]-	51.10
PG 18:3_20:4	3.55	791.4789	[M-H]-	12.93
PG 18:3_20:5	3.33	789.4758	[M-H]-	5.50
PG 18:1_22:6	3.85	819.5136	[M-H]-	32.13
PG 18:3_22:5	3.54	817.4931	[M-H]-	8.37
PG 18:3_22:6	3.47	815.4818	[M-H]-	9.37
PG 20:4_22:6	3.53	841.4950	[M-H]-	21.18
PG 20:5_22:6	3.31	839.4935	[M-H]-	5.68
PG 20:1_22:6	3.98	847.5376	[M-H]-	31.05
PG 20:3_22:5	3.77	845.5225	[M-H]-	15.05
PG 22:5_22:6	3.63	867.5137	[M-H]-	71.97
PG 22:6_22:6	3.46	865.5025	[M-H]-	55.27
PI 16:0_16:1	3.82	807.4940	[M-H]-	448.42
PI 16:0_18:1	4.14	835.5364	[M-H]-	1023.31
PI 18:0_18:3	3.91	859.5309	[M-H]-	135.73
PI 16:0_20:4	3.79	857.5211	[M-H]-	1730.84
PI 16:1_20:4	3.60	855.4996	[M-H]-	632.49
PI 17:0_20:4	3.94	871.5323	[M-H]-	1059.52
PI 17:0_20:5	3.74	869.5179	[M-H]-	274.55

PI 18:0_20:4	4.07	885.5524	[M-H]-	18567.74
PI 18:0_20:5	3.88	883.5356	[M-H]-	6428.56
PI 18:1_20:5	3.66	881.5176	[M-H]-	1037.99
PI 18:3_20:4	3.44	879.4960	[M-H]-	83.63
PI 19:0_20:4	4.20	899.5694	[M-H]-	702.49
PI 19:1_20:4	3.98	897.5482	[M-H]-	269.91
PI 17:0_22:6	3.85	895.5305	[M-H]-	161.59
PI 18:0_22:5	4.11	911.5634	[M-H]-	397.24
PI 18:0_22:6	4.01	909.5460	[M-H]-	954.87
PI 18:1_22:6	3.77	907.5294	[M-H]-	263.31
PI 20:4_20:4	3.55	905.5186	[M-H]-	150.32
PI 20:4_22:5	3.65	931.5289	[M-H]-	50.82
PS 16:0_16:1	3.85	732.4840	[M-H]-	99.86
PS 16:0_18:1	4.15	760.5121	[M-H]-	626.38
PS 16:0_18:2	3.93	758.4878	[M-H]-	94.31
PS 18:0_17:1	4.31	774.5278	[M-H]-	315.40
PS 18:0_18:1	4.49	788.5400	[M-H]-	1153.65
PS 18:1_18:1	4.23	786.5256	[M-H]-	526.57
PS 16:0_20:3	4.00	784.5099	[M-H]-	124.39
PS 16:0_20:4	3.88	782.4926	[M-H]-	224.97
PS 19:0_18:1	4.74	802.5540	[M-H]-	87.26
PS 18:0_20:3	4.34	812.5427	[M-H]-	286.53
PS 18:0_20:4	4.18	810.5260	[M-H]-	974.45
PS 18:1_20:4	4.00	808.5203	[M-H]-	915.57
PS 16:1_22:6	3.57	804.4780	[M-H]-	38.47
PS 17:0_22:6	3.97	820.5065	[M-H]-	264.17
PS 18:1_22:6	3.88	832.5073	[M-H]-	202.03
PS 22:6_22:6	3.57	878.4971	[M-H]-	401.27
PE P-16:0_14:0	5.11	648.4909	$[M+H]^+$	51.25
PE P-14:0_16:1	4.70	646.4683	$[M+H]^+$	20.98
PE P-16:0_15:1	5.02	660.4854	$[M+H]^+$	74.43
PE P-16:0_16:0	5.62	676.5159	$[M+H]^+$	168.83
PE P-16:0_17:1	5.44	688.5140	$[M+H]^+$	308.36
PE P-17:1_16:1	5.04	686.5071	$[M+H]^+$	45.17
PE P-16:0_18:1	5.71	702.5317	$[M+H]^+$	2001.43
PE P-14:0_20:5	4.44	694.4667	$[M+H]^+$	33.20
PE P-18:1_17:1	5.49	714.5358	$[M+H]^+$	242.00
PE P-15:0_20:4	4.95	710.5044	$[M+H]^+$	180.86
PE P-18:0_18:1	6.28	730.5637	$[M+H]^+$	1621.36
PE P-18:1_18:1	5.75	728.5488	$[M+H]^+$	1456.33
PE P-18:1_18:2	5.35	726.5364	$[M+H]^+$	259.95
PE P-16:0_20:4	5.18	724.5169	$[M+H]^+$	1114.76
PE P-16:0_20:5	4.88	722.5057	$[M+H]^+$	1171.89
PE P-14:0_22:6	4.61	720.4899	$[M+H]^+$	139.02

PE P-17:0_20:4	5.38	738.5336	$[M+H]^+$	335.17
PE P-17:0_20:5	5.05	736.5225	$[M+H]^+$	331.95
PE P-15:0_22:6	4.83	734.5082	$[M+H]^+$	226.87
PE P-18:1_20:1	6.30	756.5807	$[M+H]^+$	357.84
PE P-18:0_20:4	5.72	752.5516	$[M+H]^+$	684.48
PE P-16:0_22:5	5.24	750.5324	$[M+H]^+$	812.19
PE P-16:0_22:6	5.08	748.5215	$[M+H]^+$	3587.13
PE P-16:1_22:6	4.61	746.5040	$[M+H]^+$	332.24
PE P-17:0_22:6	5.27	762.5386	$[M+H]^+$	582.64
PE P-17:1_22:6	4.89	760.5205	$[M+H]^+$	126.31
PE P-18:0_22:6	5.58	776.5545	$[M+H]^+$	1065.77
PE P-18:1_22:6	5.12	774.5372	$[M+H]^+$	1166.38
PE P-18:2_22:6	4.79	772.5206	$[M+H]^+$	287.33
PE P-18:3_22:6	4.50	770.5032	$[M+H]^+$	208.77

Table S3. PL Molecular species of S-PLE determined by (ultrahigh-performance liquid chromatography-quadrupole-time-off light mass spectrometry) UHPLC-Q-TOF-MS.

Molecular Species	RT (min)	m/z	Precursors	Contents/(nmol/g)
LPA 18:2	2.21	433.2300	[M-H]-	76.61
LPA 20:5	1.68	455.2204	[M-H]-	69.65
LPA 22:6	1.16	481.2344	[M-H]-	790.84
LPC 14:0	1.77	526.3149	[M+CH3COO]-	2098.19
LPC 16:0	2.48	554.3458	[M+CH3COO]-	20033.75
LPC 18:0	3.09	582.3763	[M+CH3COO]-	3375.09
LPC 18:1	2.55	580.3614	[M+CH3COO]-	10249.71
LPC 18:2	2.19	578.3398	[M+CH3COO]-	3360.88
LPC 18:3	1.74	576.3292	[M+CH3COO]-	1070.33
LPC 18:4	1.42	574.3089	[M+CH3COO]-	270.12
LPC 20:0	3.72	610.3994	[M+CH3COO]-	87.52
LPC 20:1	3.15	608.3893	[M+CH3COO]-	325.75
LPC 20:3	2.39	604.3613	[M+CH3COO]-	668.54
LPC 20:4	2.12	602.3474	[M+CH3COO]-	1735.14
LPC 20:5	1.68	600.3303	[M+CH3COO]-	13449.97
LPC 22:5	2.23	628.3563	[M+CH3COO]-	5995.20
LPC 22:6	2.03	626.3451	[M+CH3COO]-	81840.42
LPC 24:1	4.35	664.4512	[M+CH3COO]-	378.30
LPE 14:0	1.63	424.2480	[M-H]-	40.81
LPE 16:0	2.33	452.2770	[M-H]-	4664.71
LPE 16:1	1.86	450.2532	[M-H]-	53.59
LPE 17:0	2.62	466.2870	[M-H]-	185.38
LPE 17:1	2.19	464.2734	[M-H]-	165.96
LPE 18:0	2.88	480.3075	[M-H]-	4524.72
LPE 18:1	2.46	478.2916	[M-H]-	4767.13
LPE 18:2	2.05	476.2775	[M-H]-	770.79
LPE 18:3	1.64	474.2601	[M-H]-	111.35
LPE 19:0	3.16	494.3239	[M-H]-	65.64
LPE 20:0	3.40	508.3358	[M-H]-	76.54
LPE 20:1	2.96	506.3188	[M-H]-	277.80
LPE 20:2	2.62	504.3045	[M-H]-	289.36
LPE 20:3	2.26	502.2889	[M-H]-	273.51
LPE 20:4	1.99	500.2744	[M-H]-	559.80
LPE 20:5	1.58	498.2612	[M-H]-	991.96
LPE 22:2	3.10	532.3354	[M-H]-	18.89
LPE 22:4	2.48	528.3011	[M-H]-	117.25
LPE 22:5	2.15	526.2930	[M-H]-	1657.31
LPE 22:6	1.92	524.2795	[M-H]-	15600.17
LPG 14:0	1.10	455.2395	[M-H]-	42.41
LPG 16:0	1.33	483.2716	[M-H]-	2095.79
LPG 18:0	2.03	511.2969	[M-H]-	614.67

LPG 18:1	1.49	509.2874	[M-H]-	3427.99
LPG 18:2	1.12	507.2692	[M-H]-	630.73
LPG 20:1	2.15	537.3173	[M-H]-	167.04
LPG 20:4	1.10	531.2701	[M-H]-	227.41
LPG 20:5	0.90	529.2548	[M-H]-	72.07
LPG 22:5	1.19	557.2852	[M-H]-	704.02
LPG 22:6	1.09	555.2722	[M-H]-	6412.68
LPG 24:1	3.01	593.3796	[M-H]-	171.12
LPG O-16:0	1.65	469.2893	[M-H]-	129.69
LPG O-18:2	1.77	493.2897	[M-H]-	203.92
LPI 18:2	1.09	595.2872	[M-H]-	108.68
LPI 20:4	1.01	619.2910	[M-H]-	413.78
LPI 20:5	0.84	617.2741	[M-H]-	83.77
LPS 16:0	1.28	496.2672	[M-H]-	478.17
LPS 18:1	1.40	522.2808	[M-H]-	1632.14
LPS 18:2	1.15	520.2679	[M-H]-	170.06
LPS 20:1	2.08	550.3055	[M-H]-	181.94
LPS 20:3	1.24	546.2781	[M-H]-	139.52
LPS 20:4	1.12	544.2653	[M-H]-	256.52
LPS 20:5	0.87	542.2515	[M-H]-	38.45
LPS 22:5	1.24	570.2827	[M-H]-	1575.62
LPS 22:6	1.15	568.2687	[M-H]-	7568.90
PA 16:0_18:1	4.34	673.4770	[M-H]-	239.42
PA 16:0_18:2	4.08	671.4652	[M-H]-	60.92
PA 18:0_20:4	4.25	723.5005	[M-H]-	81.71
PA 16:0_22:6	3.83	719.4739	[M-H]-	720.25
PA 18:0_22:6	4.31	747.4926	[M-H]-	1110.97
PA 20:4_20:4	3.76	743.4545	[M-H]-	205.36
PC 16:0_16:0	5.93	792.5763	[M+CH3COO]-	3320.96
PC 16:0_18:1	5.97	818.5922	[M+CH3COO]-	116692.23
PC 16:0_18:2	5.56	816.5797	[M+CH3COO]-	12574.37
PC 16:0_18:3	5.15	814.5574	[M+CH3COO]-	1202.78
PC 17:0_18:1	6.54	832.6074	[M+CH3COO]-	1973.15
PC 18:0_18:1	6.87	846.6245	[M+CH3COO]-	26892.99
PC 18:1_18:1	6.11	844.6060	[M+CH3COO]-	8127.47
PC 18:1_18:2	5.61	842.5929	[M+CH3COO]-	4477.87
PC 18:1_18:3	5.17	840.5740	[M+CH3COO]-	1467.17
PC 16:0_20:5	5.03	838.5645	[M+CH3COO]-	17850.60
PC 19:0_18:1	7.37	860.6387	[M+CH3COO]-	479.56
PC 15:0_22:6	4.98	850.5586	[M+CH3COO]-	276.62
PC 18:1_20:2	6.33	870.6156	[M+CH3COO]-	835.02
PC 16:0_22:5	5.46	866.5967	[M+CH3COO]-	4996.41
PC 18:0_20:5	5.61	866.5914	[M+CH3COO]-	4912.19
PC 16:0_22:6	5.21	864.5740	[M+CH3COO]-	53808.04

PC 17:0_22:6	5.55	878.5912	[M+CH3COO]-	332.07
PC 22:0_18:1	8.82	902.6841	[M+CH3COO]-	938.08
PC 18:1_22:1	7.83	900.6726	[M+CH3COO]-	2356.97
PC 18:1_22:2	7.14	898.6517	[M+CH3COO]-	699.09
PC 18:0_22:5	6.16	894.6140	[M+CH3COO]-	506.58
PC 18:0_22:6	5.85	892.6071	[M+CH3COO]-	3284.05
PC 18:2_22:6	4.91	888.5767	[M+CH3COO]-	2523.11
PC 23:0_18:1	9.42	916.6964	[M+CH3COO]-	301.28
PC 18:1_23:1	8.34	914.6857	[M+CH3COO]-	498.81
PC 24:0_18:1	9.94	930.7206	[M+CH3COO]-	2611.57
PC 20:5_22:6	4.48	910.5539	[M+CH3COO]-	2029.12
PC 18:1_24:1	8.69	928.6996	[M+CH3COO]-	33439.01
PC 18:2_24:2	7.35	924.6635	[M+CH3COO]-	688.22
PC 22:0_20:5	7.15	922.6484	[M+CH3COO]-	332.83
PC 22:1_20:5	6.37	920.6316	[M+CH3COO]-	232.66
PC 20:1_22:6	5.85	918.6214	[M+CH3COO]-	264.66
PC 22:5_22:6	4.85	938.5799	[M+CH3COO]-	568.09
PC 22:6_22:6	4.67	936.5760	[M+CH3COO]-	3098.45
PC 18:1_26:1	9.86	956.7296	[M+CH3COO]-	2901.00
PC 18:1_26:2	9.02	954.7147	[M+CH3COO]-	1039.19
PC 24:1_20:3	8.24	952.6949	[M+CH3COO]-	481.92
PC 24:0_20:5	8.07	950.6867	[M+CH3COO]-	729.49
PC 24:1_20:4	7.68	950.6816	[M+CH3COO]-	394.82
PC 24:1_20:5	7.14	948.6682	[M+CH3COO]-	2792.92
PC 22:1_22:6	6.63	946.6545	[M+CH3COO]-	321.21
PC 24:1_22:5	7.79	976.6980	[M+CH3COO]-	569.26
PC 24:1_22:6	7.40	974.6873	[M+CH3COO]-	1925.88
PC O-16:0_16:1;3O	5.46	824.5770	[M+CH3COO]-	2159.54
PC O-16:0_18:2;3O	5.69	850.5958	[M+CH3COO]-	20039.21
PC O-18:1_18:2;3O	5.76	876.6057	[M+CH3COO]-	2217.40
PE 16:0_16:0	5.35	690.5051	[M-H]-	169.47
PE 14:0_18:1	4.92	688.4900	[M-H]-	194.97
PE 16:0_18:1	5.40	716.5203	[M-H]-	4471.86
PE 16:0_18:2	5.03	714.5051	[M-H]-	780.69
PE 16:0_18:3	4.73	712.4864	[M-H]-	107.34
PE 14:0_20:4	4.51	710.4673	[M-H]-	19.49
PE 18:0_17:1	5.71	730.5356	[M-H]-	432.51
PE 17:1_18:1	5.25	728.5197	[M-H]-	324.84
PE 17:1_18:2	4.90	726.5030	[M-H]-	87.71
PE 18:0_18:1	5.96	744.5580	[M-H]-	14889.87
PE 18:1_18:1	5.48	742.5372	[M-H]-	8738.53
PE 18:1_18:2	5.10	740.5214	[M-H]-	1436.47
PE 16:0_20:4	4.95	738.5041	[M-H]-	901.23
PE 16:0_20:5	4.67	736.4937	[M-H]-	2156.65

PE 14:0_22:6	4.42	734.4711	[M-H]-	117.22
PE 18:0_19:1	6.54	758.5656	[M-H]-	130.47
PE 18:1_19:1	5.82	756.5562	[M-H]-	81.84
PE 18:1_20:1	6.00	770.5707	[M-H]-	3526.43
PE 18:1_20:2	5.58	768.5526	[M-H]-	1963.16
PE 18:0_20:4	5.45	766.5380	[M-H]-	4260.05
PE 18:0_20:5	5.12	764.5214	[M-H]-	4380.37
PE 18:1_20:4	5.01	764.5211	[M-H]-	3918.64
PE 16:0_22:6	4.85	762.5101	[M-H]-	21078.32
PE 18:2_20:5	4.42	760.4913	[M-H]-	487.45
PE 17:0_22:6	5.08	776.5198	[M-H]-	441.70
PE 18:0_22:1	7.15	800.6124	[M-H]-	50.57
PE 18:1_22:1	6.59	798.5992	[M-H]-	160.05
PE 18:1_22:2	6.10	796.5765	[M-H]-	248.16
PE 18:0_22:4	5.87	794.5646	[M-H]-	417.14
PE 18:0_22:5	5.49	792.5572	[M-H]-	3271.43
PE 18:0_22:6	5.33	790.5388	[M-H]-	22340.94
PE 18:1_22:6	4.91	788.5237	[M-H]-	10078.83
PE 18:2_22:6	4.59	786.5101	[M-H]-	2923.31
PE 18:3_22:6	4.32	784.4842	[M-H]-	457.22
PE 20:5_20:5	4.12	782.4785	[M-H]-	61.50
PE 19:0_22:6	5.59	804.5564	[M-H]-	254.99
PE 19:1_22:6	5.12	802.5320	[M-H]-	85.58
PE 18:1_24:1	7.18	826.6336	[M-H]-	588.20
PE 18:1_24:2	6.69	824.6163	[M-H]-	268.46
PE 20:2_22:6	5.00	814.5392	[M-H]-	685.53
PE 20:3_22:6	4.70	812.5246	[M-H]-	783.79
PE 20:4_22:6	4.42	810.5088	[M-H]-	609.48
PE 20:5_22:6	4.27	808.4918	[M-H]-	725.64
PE 22:5_22:6	4.56	836.5235	[M-H]-	1651.64
PE 22:6_22:6	4.42	834.5072	[M-H]-	2378.80
PE 24:1_20:3	7.35	850.6274	[M-H]-	83.15
PE O-16:1_16:0	5.65	674.5074	[M-H]-	122.67
PE O-16:1_16:1	5.21	672.4941	[M-H]-	444.25
PE O-16:1_17:1	5.49	686.5055	[M-H]-	332.95
PE O-18:1_16:0	6.23	702.5439	[M-H]-	290.10
PE O-16:1_18:1	5.71	700.5267	[M-H]-	9414.88
PE O-18:2_16:1	5.28	698.5125	[M-H]-	2237.06
PE O-16:1_18:3	4.98	696.4976	[M-H]-	189.06
PE O-18:1_17:1	6.04	714.5447	[M-H]-	1040.01
PE O-18:2_17:1	5.55	712.5299	[M-H]-	1271.25
PE O-18:0_18:1	6.75	730.5728	[M-H]-	736.88
PE O-18:1_18:1	6.30	728.5608	[M-H]-	12341.23
PE O-18:2_18:1	5.78	726.5430	[M-H]-	23402.21

PE O-18:3_18:1	5.38	724.5247	[M-H]-	3351.68
PE O-16:1_20:4	5.21	722.5137	[M-H]-	1106.55
PE O-16:1_20:5	4.90	720.4927	[M-H]-	1331.11
PE O-14:1_22:6	4.64	718.4802	[M-H]-	34.66
PE O-19:2_18:1	6.06	740.5543	[M-H]-	197.63
PE O-17:1_20:4	5.46	736.5198	[M-H]-	80.74
PE O-17:1_20:5	5.11	734.5032	[M-H]-	127.49
PE O-15:1_22:6	4.85	732.4880	[M-H]-	62.85
PE O-20:0_18:1	7.71	758.5966	[M-H]-	110.23
PE O-18:1_20:1	6.89	756.5923	[M-H]-	590.23
PE O-18:2_20:1	6.34	754.5731	[M-H]-	793.12
PE O-20:3_18:1	5.89	752.5543	[M-H]-	568.23
PE O-18:1_20:4	5.73	750.5429	[M-H]-	1212.92
PE O-18:2_20:4	5.26	748.5296	[M-H]-	6065.92
PE O-18:2_20:5	4.95	746.5121	[M-H]-	3427.71
PE O-18:3_20:5	4.62	744.4971	[M-H]-	216.66
PE O-17:1_22:6	5.28	760.5168	[M-H]-	326.74
PE O-17:2_22:6	4.91	758.5045	[M-H]-	28.92
PE O-18:1_22:1	7.52	784.6169	[M-H]-	75.06
PE O-18:2_22:1	6.95	782.6055	[M-H]-	229.73
PE O-18:2_22:2	6.45	780.5869	[M-H]-	190.48
PE O-18:0_22:6	5.69	776.5579	[M-H]-	3049.14
PE O-18:1_22:6	5.61	774.5468	[M-H]-	2058.93
PE O-18:2_22:5	5.31	774.5417	[M-H]-	1342.79
PE O-18:2_22:6	5.15	772.5302	[M-H]-	12145.94
PE O-18:3_22:6	4.80	770.5135	[M-H]-	539.65
PG 14:0_16:0	3.77	693.4619	[M-H]-	35.74
PG 14:0_16:1	3.54	691.4554	[M-H]-	10.93
PG 14:0_18:2	3.60	717.4652	[M-H]-	18.33
PG 15:0_18:1	3.95	733.4955	[M-H]-	28.15
PG 16:0_18:1	4.08	747.5186	[M-H]-	2194.38
PG 16:0_18:2	3.88	745.5023	[M-H]-	297.85
PG 16:0_18:3	3.70	743.4830	[M-H]-	86.98
PG 14:0_20:4	3.57	741.4695	[M-H]-	28.30
PG 14:0_20:5	3.37	739.4510	[M-H]-	19.02
PG 17:1_18:1	4.00	759.5191	[M-H]-	28.03
PG 18:1_17:2	3.84	757.4971	[M-H]-	28.04
PG 18:0_18:1	4.39	775.5450	[M-H]-	215.16
PG 18:1_18:1	4.14	773.5311	[M-H]-	474.05
PG 18:1_18:2	3.94	771.5162	[M-H]-	375.99
PG 16:0_20:4	3.86	769.4964	[M-H]-	122.54
PG 16:0_20:5	3.69	767.4876	[M-H]-	613.63
PG 14:0_22:6	3.53	765.4662	[M-H]-	56.91
PG 18:1_20:1	4.42	801.5617	[M-H]-	37.37

PG 18:0_20:4	4.14	797.5322	[M-H]-	88.19
PG 18:0_20:5	3.95	795.5184	[M-H]-	321.87
PG 16:0_22:6	3.81	793.4979	[M-H]-	1837.50
PG 16:1_22:6	3.55	791.4869	[M-H]-	137.05
PG 18:3_20:5	3.31	789.4649	[M-H]-	23.35
PG 18:1_22:1	4.77	829.5984	[M-H]-	22.22
PG 22:1_18:2	4.51	827.5771	[M-H]-	13.12
PG 18:0_22:5	4.15	823.5331	[M-H]-	77.35
PG 18:0_22:6	4.08	821.5311	[M-H]-	192.32
PG 18:1_22:6	3.86	819.5164	[M-H]-	453.87
PG 18:2_22:6	3.64	817.5017	[M-H]-	320.19
PG 18:3_22:6	3.45	815.4860	[M-H]-	113.28
PG 20:5_20:5	3.29	813.4722	[M-H]-	27.97
PG 18:1_24:1	5.14	857.6246	[M-H]-	75.11
PG 18:1_24:2	4.85	855.6108	[M-H]-	53.78
PG 20:1_22:6	4.11	847.5403	[M-H]-	19.08
PG 20:3_22:6	3.73	843.5082	[M-H]-	31.75
PG 20:4_22:6	3.55	841.4974	[M-H]-	43.23
PG 20:5_22:6	3.41	839.4857	[M-H]-	75.49
PG 24:1_20:5	4.59	877.5942	[M-H]-	51.26
PG 22:5_22:6	3.63	867.5130	[M-H]-	68.21
PG 22:6_22:6	3.45	865.4977	[M-H]-	101.29
PG 24:1_22:6	4.73	903.6112	[M-H]-	93.39
PI 18:0_18:1	4.32	863.5532	[M-H]-	451.50
PI 18:0_18:2	4.10	861.5428	[M-H]-	916.06
PI 18:0_18:3	3.91	859.5308	[M-H]-	646.68
PI 16:0_20:4	3.79	857.5082	[M-H]-	499.23
PI 16:0_20:5	3.60	855.5035	[M-H]-	662.58
PI 17:0_20:4	3.94	871.5325	[M-H]-	139.58
PI 17:0_20:5	3.75	869.5150	[M-H]-	140.62
PI 18:0_20:4	4.07	885.5503	[M-H]-	11308.31
PI 18:0_20:5	3.90	883.5313	[M-H]-	9199.45
PI 16:0_22:6	3.73	881.5121	[M-H]-	1944.27
PI 18:0_22:6	4.01	909.5480	[M-H]-	9480.79
PI 18:1_22:6	3.77	907.5265	[M-H]-	464.30
PI 18:2_22:6	3.57	905.5135	[M-H]-	55.89
PI 18:3_22:6	3.37	903.4938	[M-H]-	50.84
PS 16:0_16:1	3.83	732.4833	[M-H]-	165.57
PS 16:0_18:1	4.14	760.5131	[M-H]-	1787.99
PS 16:0_18:2	3.93	758.4969	[M-H]-	331.15
PS 16:0_18:3	3.76	756.4901	[M-H]-	72.35
PS 18:0_18:1	4.47	788.5557	[M-H]-	977.35
PS 18:1_18:1	4.21	786.5291	[M-H]-	1820.32
PS 18:1_18:2	3.97	784.5103	[M-H]-	376.04

PS 18:0_20:3	4.35	812.5366	[M-H]-	830.77
PS 18:0_20:4	4.18	810.5255	[M-H]-	1206.61
PS 18:1_20:4	3.97	808.5118	[M-H]-	2672.25
PS 18:2_22:6	3.66	830.4908	[M-H]-	358.38
PS 20:1_22:6	4.14	860.5317	[M-H]-	178.02
PS 20:3_22:6	3.73	856.5109	[M-H]-	55.77
PS 20:5_22:6	3.41	852.4750	[M-H]-	191.17
PS 22:5_22:6	3.66	880.5156	[M-H]-	591.39
PS 22:6_22:6	3.55	878.4944	[M-H]-	838.00
PE P-16:0_16:1	5.15	674.5063	[M+H]+	323.59
PE P-18:0_16:0	6.22	704.5496	[M+H]+	124.26
PE P-16:0_18:1	5.69	702.5345	[M+H]+	3711.20
PE P-18:1_16:1	5.28	700.5186	[M+H]+	1203.06
PE P-16:0_18:3	4.96	698.5052	[M+H]+	300.40
PE P-18:1_17:1	5.52	714.5347	[M+H]+	714.10
PE P-18:0_18:1	6.28	730.5673	[M+H]+	4389.85
PE P-18:1_18:1	5.76	728.5520	[M+H]+	9711.33
PE P-18:1_18:2	5.35	726.5364	[M+H]+	2304.03
PE P-16:0_20:4	5.18	724.5192	[M+H]+	527.20
PE P-16:0_20:5	4.88	722.5059	[M+H]+	2043.38
PE P-14:0_22:6	4.61	720.4905	[M+H]+	101.51
PE P-15:0_22:6	4.84	734.5046	[M+H]+	105.00
PE P-18:0_20:1	6.87	758.5972	[M+H]+	200.51
PE P-18:1_20:1	6.32	756.5801	[M+H]+	489.83
PE P-18:1_20:2	5.86	754.5692	[M+H]+	290.13
PE P-18:1_20:4/18:0_20:5	5.25	750.5341	[M+H]+	1543.41
PE P-16:0_22:6	5.07	748.5243	[M+H]+	12569.40
PE P-17:0_22:6	5.31	762.5359	[M+H]+	195.75
PE P-18:0_22:5	5.77	778.5682	[M+H]+	532.68
PE P-18:0_22:6	5.58	776.5552	[M+H]+	897.65
PE P-18:1_22:6	5.13	774.5397	[M+H]+	7778.65
PE P-18:2_22:6	4.78	772.5233	[M+H]+	1107.60
